# Immunization knowledge and practice among Malaysian parents: a questionnaire development and pilot-testing

**DOI:** 10.1186/1471-2458-14-1107

**Published:** 2014-10-27

**Authors:** Ammar Ihsan Awadh, Mohamed Azmi Hassali, Omer Qutaiba Al-lela, Siti Halimah Bux, Ramadan M Elkalmi, Hazrina Hadi

**Affiliations:** Department of Pharmacy Practice, Kulliyyah of Pharmacy, International Islamic University Malaysia, 25200 Kuantan, Malaysia; Discipline of Social and Administrative Pharmacy, School of Pharmaceutical Sciences, Universiti Sains Malaysia, 11800 Penang, Malaysia; School of Pharmacy, Faculty of Medical sciences, University of Duhok (UOD), Duhok, Iraq; Department of Pharmaceutical Technology, Kulliyyah of Pharmacy, International Islamic University Malaysia, 25200 Kuantan, Malaysia

**Keywords:** Immunization, Reliability, Validity, Questionnaire development, Knowledge, Practice

## Abstract

**Background:**

Parents are the main decision makers for their children vaccinations. This fact makes parents’ immunization knowledge and practices as predictor factors for immunization uptake and timeliness. The aim of this pilot study was to develop a reliable and valid instrument in Malaysian language to measure immunization knowledge and practice (KP) of Malaysian parents.

**Methods:**

A cross-sectional prospective pilot survey was conducted among 88 Malaysian parents who attended public health facilities that provide vaccinations. Translated immunization KP questionnaires (Bahasa Melayu version) were used. Descriptive statistics were applied, face and content validity were assessed, and internal consistency, test-retest reliability, and construct validity were determined.

**Results:**

The mean ± standard deviation (SD) of the knowledge scores was 7.36 ± 2.29 and for practice scores was 7.13 ± 2.20. Good internal consistency was found for knowledge and practice items (Cronbach’s alpha = 0.757 and 0.743 respectively); the test-retest reliability value was 0.740 (*p* = 0.014). A panel of three specialist pharmacists who are experts in this field judged the face and content validity of the final questionnaire. Parents with up-to-date immunized children had significantly better knowledge and practice scores than parents who did not (*p* < 0.001 and *p* = 0.001 respectively), suggesting a good construct validity. A significant difference was found in knowledge and practice scores among parents’ age (*p* = 0.006 and *p* = 0.029 respectively) and place of living (*p* = 0.037 and *p* = 0.043). The parents’ knowledge level was positively associated with their practice toward immunization (Spearman’s rank correlation coefficient 0.310, *p* = 0.003).

**Conclusions:**

The pilot study concluded that the Bahasa Melayu version of the immunization KP questionnaire has good reliability and validity for measuring the knowledge and practices of Malaysian parents and therefore this version can be used in future research.

## Background

In health care, prevention of disease is always better than cure. It is undeniable that vaccines have been an important part in preventive medicine based on their successes in controlling vaccine-preventable diseases in the developed world. The eradication, elimination, and significant reduction in the number of cases of childhood communicable diseases as well as the fact that they extend life expectancy in the United States and many other parts of the world are some of the examples of immunizations’ success in the 20th century [1].

Ehreth estimated that annually up to three million children’s lives are saved by vaccinations, but still another three million lives worldwide are lost from vaccine-preventable diseases [2]. Furthermore, the advantages of vaccinations extend beyond just preventing specific diseases; they can prevent the development of antibiotic resistance, empower women, protect against bioterrorism, ensure safe travel and mobility, promote economic growth, enhance equity, promote peace, and extend life expectancy [3].

In Malaysia, immunization program was introduced in early 1950s and vaccines are provided free of charge in public health facilities nationwide. Impressive child immunization levels is one of the aspects that has contributed to Malaysian children’s good health status [4]. Despite such global success and the proven impact on human health and life expectancy, vaccines are still under-utilized [5]. One hundred thirty million children are born annually, but around 30 million of them still have no access to vaccinations [2]. Low levels of compliance in a variety of settings have been clearly documented [6, 7].

There are several factors that can lead to inadequate immunization. Studies have shown that improving parents’ knowledge regarding vaccines improves immunization status and affects the success of immunization programs [8, 9]. One of the major barriers towards children’s immunizations is parental misperceptions toward immunizations [10]. Parents’ misperceptions can lead to the suboptimal use of vaccines. In London, from 1996 to 2001, substantial measles outbreaks remained rare, occurring primarily because small clusters of parents had misguided safety concerns about vaccinations [11].

Because parents are the primary health decision-makers for their children, their knowledge and practices regarding immunization in general have a great impact on the immunization status of their children [12, 13]. The huge amount of conflicting vaccine-safety information and misinformation on the Internet can negatively influence parents’ decisions [14]. Thus, there is a vital need to assess parents’ knowledge and practice regarding their children immunization in order to improve and increase vaccination coverage and completeness.

Much has been published about parents’ knowledge and practices regarding childhood immunizations [8, 15–17]. However, no studies have been reported in Malaysia. This study was conducted to evaluate the knowledge and practices of Malaysian parents about their children’s immunizations.

### Study objectives

The main objectives of this study were to develop a questionnaire to measure immunization knowledge and practices (KP) among Malaysian parents, to translate the developed instrument into a Malay version, and to determine the validity and reliability of the developed translated instrument, and to assess differences in parents’ KP across their socio-demographic characteristics. The questionnaire will help to identify the relationship between immunization KP of parents and the immunization status of their children.

## Methods

### Development of the questionnaire

The questionnaire was developed after an extensive literature search. The original questionnaire was developed in the English language in order to maintain consistency with the questions adapted from the references with and without modifications [1, 5, 18–21]. Additional questions were added to cover the objectives of this study. The final questionnaire consisted of 20 questions: 10 questions regarding knowledge with a "yes/no/don't know" answer format and 10 questions about practices with a "yes/no" answer format.

### Translation

The questionnaire was translated into the Malaysian language (Bahasa Melayu) according to the recommended procedures and guidelines by Guillemin and Beaton [22, 23]. In the first step, two different translators who speak both English and Bahasa Melayu, but whose native language was Bahasa Melayu, translated the questionnaire from English into Bahasa Melayu. In order to enhance the quality of the translation, one of the two translators was familiar with the aims of the questionnaire in this study whereas the other was not. One of the researchers, who is Malaysian-Malay, reviewed the two primary versions and compared them to the original; then, the first version of the questionnaire was prepared.

The second step involved the reverse translation of the questionnaire from the first version of the Bahasa Melayu questionnaire into English. The reverse translation was carried out by two other translators who were fluent in both English and Bahasa Melayu but who did not know the aims of this questionnaire. The result of the reverse translation was compared to the original English questionnaire, and the second report was prepared. Repeated discussions between all of the translators and the researchers were carried out in order to ensure the accuracy of the questionnaire.

The final stage was a pilot test of the questionnaire. Ten Malaysian parents completed the questionnaire and commented on the questions. These ten parents were not included in the study. Both the first report and the second report and the parents’ comments were discussed by the researchers and necessary amendments were made accordingly. The final KP Questionnaire (Bahasa Melayu) version on immunization was done and made ready for the validity and reliability study.

### Data collection

The data collection form consisted of two parts. The first part contained parents’ socio-demographic data. The second part consisted of the knowledge and practice questionnaire (10 questions on knowledge and 10 questions on practice).

Parents who were visiting the clinic for any reason, had a child aged at least two years old (to ensure the completeness of immunization histories for the first two years of the child age), and not more than five years old, and lived in Pahang were included in this study. Parents who do not have a child aged at least two years old, and not more than five years old and, lived outside Pahang were excluded.

The data were collected from ten public clinics in five districts (Bera, Kuantan, Maran, Pekan, and Temerloh) across state of Pahang, the largest state in Peninsular Malaysia with an area of 36.173 km^2^ and population of 1,443,365 (2010 census). Eighty-eight Malaysian parents who attended public health facilities that provide vaccinations (a health clinic, a community clinic, and a maternal and child health clinic) were included in this study. For the pilot study, the recommended sample size was fewer than 100 subjects [24]. However, there were only 88 parents who fulfilled the study criteria.

Ethical approval for the study was obtained from the Medical Research Ethic Committee (MREC). All parents in the sample read a cover letter describing the study objectives and the time needed to fill in the questionnaire and received written informed consent for their participation before involving them in the research. The final translated version of the knowledge and practices questionnaire regarding childhood immunization was given to the parents.

### Validity and reliability study

A panel of three specialist pharmacists who are experts in this field subsequently discussed and judged the face and content validity of the final questionnaire. The questions were modified as necessary to reflect the results of the pre-testing. To assess the reliability of the questionnaire, internal consistency reliability testing was conducted and test-retest reliability was conducted by asking a subgroup of these parents (12 parents) after a two-week period to complete a second copy of the questionnaire. A known-group validity technique was used to determine the construct validity of the questionnaire by assessing the association between parents’ KP and the immunization status of their children (Up-to-Date and Not Up-to-Date).

### Statistical analysis

The data were tabulated and analyzed using Microsoft Excel and the Statistical Package for Social Sciences SPSS 20 for Windows. The frequency and the percentage of each demographic data parameter, including age, gender, race, religion, number of pre-school children, family size, place of living, employment status, educational level, and family income were evaluated. The means and standard deviations were calculated for total knowledge score and total practice score, and they were treated as continuous variables. Scoring of the questions was determined by giving one point (1) for each correct answer and zero (0) for incorrect answers or for no response.

Mean and median for the total knowledge score and total practice score were calculated. The maximum possible score was 10 for each. Kruskal-Wallis and Mann- Whitney tests were used to determine the differences among groups for non-parametric distribution. Reliability was tested for internal consistency and corrected item-total correlations using Cronbach’s alpha coefficient. Spearman’s rank correlation coefficient was used to obtain test-retest reliability values [24]. Known group validity was assessed through the association of immunization status (Up-to-Date and Not Up-to-Date), total knowledge score, and total practice score by using the Mann–Whitney U test.

## Results

### Characteristics of parents

A total of 88 parents participated in this study. The vast majority of the respondent parents were mothers 92.0% (n = 81). The majority of the parents were between 20 and 40 years old 77.3% (n = 68). The vast majority of the parents were Malay 76.1% (n = 67). Most of the parents had a secondary education 68.2% (n = 60) (Table 1).

**Table 1:** **Socio-demographic characteristics of parents**

Parameters	Frequency (n = 88)	%
**Gender**		
Male	7	8.0
Female	81	92.0
**Age**		
<20	5	5.7
20-30	36	40.9
30-40	32	36.4
>40	15	17.0
**Race**		
Malay	67	76.1
Chinese	16	18.2
Indian	3	3.4
Others	2	2.3
**Religion**		
Islam	68	77.3
Buddhism	16	18.2
Hinduism	3	3.4
Others	1	1.1
**No. of preschool children**		
1-2	73	83
>2	15	17
**Family size**		
<4	31	35.2
4-6	45	51.1
>6	12	13.6
**Place of living**		
Rural	38	43.2
Urban	50	56.8
**Employment status**		
Employed	47	53.4
Unemployed	41	46.6
**Educational level**		
Primary	7	8.0
Secondary	60	68.2
Tertiary education	21	23.9
**Family income**		
<RM 1000	19	21.6
RM1000-RM2000	27	30.7
RM2001-RM3000	19	21.6
RM3001-RM4000	10	11.4
>RM4001	13	14.8

### Reliability

#### Internal consistency

The mean ± standard deviation for the total knowledge score and total practice score were 7.63 ± 2.29 and 7.13 ± 2.20, respectively. Internal consistency was determined for the 10 items of knowledge on the questionnaire and 10 items of practice on the questionnaire with Cronbach’s value of 0.739 and 0.732, respectively which indicate good reliability of both instruments.

The item-total correlations between the 10 items of knowledge on the questionnaire and 10 items of practice on the questionnaire are presented in Tables 2 and 3.

**Table 2:** **Reliability test of 10 items of the knowledge scale**

Question no.	Corrected item-total correlation	Cronbach's Alpha if item deleted
Q1	0.277	0.739
Q2	0.570	0.698
Q3	0.522	0.700
Q4	0.385	0.720
Q5	0.400	0.718
Q6	0.336	0.729
Q7	0.570	0.698
Q8	0.255	0.743
Q9	0.511	0.701
Q10	0.316	0.729

**Table 3:** **Reliability test of 10 items of the practice scale**

Question no.	Corrected item-total correlation	Cronbach's Alpha if item deleted
Q1	0.422	0.714
Q2	0.413	0.707
Q3	0.467	0.697
Q4	0.454	0.700
Q5	0.522	0.686
Q6	0.384	0.712
Q7	0.333	0.719
Q8	0.317	0.726
Q9	0.300	0.723
Q10	0.394	0.715

### Test-retest reliability

The investigation for the test-retest reliability with an interval of two weeks for twelve parents showed satisfactory reliability and stability with a Spearman’s rank correlation coefficient value of 0.740 (*p* < 0.05).

### Validity

#### Known-groups validity

The Mann–Whitney U test showed a significant difference between parents’ total knowledge score and their child immunization status, and between parents’ total practice score and their child immunization status (*p* < 0.001 and *p* = 0.001 respectively). Parents who had up-to-date immunized children showed higher knowledge and practice scores than parents who did not (see Table 4).

**Table 4:** **Relationship between knowledge scores and practice score and immunization status**

Parameter	Knowledge scores mean	Knowledge scores median	***P***Value	Practice scores mean	Practice scores median	***P***Value
**Immunization status**			<0.001*			0.001*
Up to Date	8.14	9.00		7.62	8.00	
Not Up to Date	6.09	6.50		5.64	6.50	

*Significant, *P*-value < 0.05.

### Parents’ knowledge and practice

The parents’ total knowledge score was positively associated with the total practice score toward immunization (Spearman’s rank correlation coefficient 0.310, *p* = 0.003).

A significant difference was found in the total knowledge scores and the total practice scores among parents’ age groups (*p* = 0.006 and *p* = 0.029 respectively) in that parents who were younger than 20 years old had lower knowledge and practice scores. A significant difference was also found in total knowledge scores and total practice scores among parents’ place of living (*p* = 0.037 and *p* = 0.043 respectively) in that parents who were living in urban areas had higher knowledge and practice scores. A significant difference was also found in total practice scores among parents’ employment status (*p* = 0.037) in that employed parents showed higher practice scores (Table 5).

**Table 5:** **KP score difference between different demographic parameters**

Parameter	Knowledge scores mean	Knowledge scores median	***P***value	Practice scores mean	Practice scores median	***P***value
**Gender**			0.095			0.505
Male	9.00	9.00		7.86	7.00	
Female	7.51	8.00		7.06	7.00	
**Age**			0.006*			0.029*
<20	5.00	5.00		7.20	7.00	
20-30	7.33	8.00		7.08	7.00	
30-40	7.94	9.00		6.50	7.00	
>40	8.53	9.00		8.53	9.00	
**Race**			0.093			0.461
Malay	7.84	9.00		7.13	7.00	
Chinese	7.63	8.00		7.50	7.00	
Indian	4.00	3.00		4.67	3.00	
Others	6.00	6.00		7.50	7.50	
**Religion**			0.067			0.286
Islam	7.84	8.50		7.18	7.00	
Buddhism	7.63	8.00		7.50	7.00	
Hinduism	4.00	3.00		4.67	3.00	
Others	4.00	4.00		5.00	5.00	
**No. of preschool children**			0.791			0.61
1-2	7.59	8.00		6.92	7.00	
3-4	7.80	9.00		8.13	8.00	
**Family size**			0.988			0.632
<4	7.61	8.00		7.42	8.00	
4-6	7.69	8.00		6.93	7.00	
>6	7.42	8.50		7.08	7.00	
**Place of living**			0.037*			0.043*
Rural	7.24	8.00		6.66	7.00	
Urban	8.13	9.00		7.48	8.00	
**Employment status**			0.140			0.031*
Employed	7.98	9.00		7.62	8.00	
Unemployed	7.22	8.00		6.56	7.00	
**Educational level**			0.611			3.03
Primary	7.86	9.00		6.00	7.00	
Secondary	7.53	8.00		7.18	7.00	
Tertiary	7.6250	8.00		7.3333	8.00	
**Family income**			0.844			0.567
< RM 1000	7.11	7.00		6.79	7.00	
RM1001-RM2000	7.74	8.00		7.19	7.00	
RM2001-RM3000	8.11	8.00		7.74	8.00	
RM3001-RM4000	7.50	8.50		6.70	7.00	
>RM4001	7.54	9.00		6.92	8.00	

*Significant, *p*-value < 0.05.

## Discussion

The aim of this study was to develop a reliable and valid questionnaire covering all aspects of parents’ immunization knowledge and practice that could be used in future studies to look at the relationship between Malaysian parents’ immunization knowledge and practices and the immunization status of their children. This is the first time a questionnaire for measuring immunization knowledge and practices of Malaysian parents has been developed and validated.

Several instruments for measuring knowledge and practices regarding immunizations have been developed in various countries at different times [20, 21, 25, 26], using these instruments and/or comparing these studies can provide interesting results but must be done with caution. Indeed, because many factors can influence parents’ knowledge, their attitudes and practices regarding immunization should be taken into account, such as their different socioeconomic characteristics, and the cultural and religious beliefs of different populations.

The developed questionnaire (in the English language) was translated into Bahasa Melayu, the common spoken language of Malaysians. The translation was carried out in accordance with the standard procedures detailed in translation guidelines by Beaton et al. [22] and Guillemin et al. [23]. The development and validation of the original items made the questionnaire relatively simple and practical to use among the Malaysian population.

The internal consistency reliability of the final questionnaire was good for both knowledge and practice (exceeded the minimum α-coefficient of 0.70). The test-retest reliability was also good. The known group validity showed parents of children who had up-to-date immunizations had significantly higher knowledge and practice scores than those who did not. This significant difference between the scores (total knowledge score/total practice score) of the parents who had up-to-date immunized children and those who did not, indicates that the questionnaire had a good construct validity.

The finding of the study indicates that the developed, translated questionnaire truly measures the immunization knowledge and practice of Malaysian parents. Moreover, the finding indicates how parents’ knowledge and practice can affect the immunization status of their children.

This study found significant positive correlations between parents’ knowledge level and their practice level; this is consistent with other studies conducted in China and in Iraq [21, 26]. This shows that parents who have adequate levels of knowledge regarding immunization also have positive practices towards immunization.

This study found significant differences in knowledge and practice scores of the parents of different age groups. Parents who were younger than 20 years old showed significantly lower knowledge and practice scores than older parents. This result is consistent with other studies that found older parents to have better knowledge and practice [27, 28]. This might be because older parents have more life experiences and better education.

This study also found that the parents’ place of living was correlated with a significant difference in the knowledge and practice scores. Parents who were living in urban areas had better knowledge and practice scores. Many studies have found that rural areas in developing countries have lower immunization coverage and age-appropriate immunization rates due to rural–urban inequities [29–31]. A significant difference between practice scores and employment status was also found. Employed parents had better practice score than unemployed parents. However, the result showed no significant differences in other demographic parameters, such as gender, race, religion, number of pre-school children, family size, educational level, or family income.

Health care professionals must be aware of parents’ knowledge and awareness of their children’s immunization to be able to design effective public education programs or measures that can help parents to make the right decisions about their children’s health and wellbeing. Those with inadequate knowledge and practices regarding immunization need to be targeted to maintain and improve immunization coverage.

### Limitations

The sample of parents was obtained from the state of Pahang (the largest state in Peninsular Malaysia) where the study was conducted, and the sample was smaller than samples used in previous studies. The findings of this pilot study may not reflect the knowledge and practice of all Malaysian parents; rather they reflect only the knowledge and practice of those who actually included in the study. Therefore, the findings need to be interpreted within the context of study limitations.

## Conclusions

This pilot study found the developed knowledge and practice questionnaire is a valid and reliable instrument for measuring Malaysian parents’ immunization knowledge and practices. This questionnaire should provide a useful tool in research on immunization knowledge and practice and allow for better understanding of the relationship between Malaysian parents’ knowledge and practices and the immunization status of their children.
